# ﻿A second species of the pill millipede genus *Nearctomeris* Wesener, 2012 (Diplopoda, Glomerida) from the Great Smoky Mountains, USA

**DOI:** 10.3897/zookeys.1166.103516

**Published:** 2023-06-13

**Authors:** Ernesto Recuero, Michael S. Caterino

**Affiliations:** 1 Department of Plant & Environmental Sciences, 277 Poole Agricultural Center, Clemson University, Clemson, SC 29634-0310, USA Clemson University Clemson United States of America

**Keywords:** Distribution, DNA barcoding, Glomeridae, integrative taxonomy, Nearctic, *Onomeris*, time-calibrated phylogeny

## Abstract

We describe a second species of *Nearctomeris* Wesener, 2012, a genus of pill millipede endemic to the southern Appalachians, based on morphological and molecular evidence. The fauna of Glomerida in America is characterized by its low diversity, and *Nearctomerissmoky***sp. nov.** is only the fifth species of the order known from the eastern United States. Our phylogenetic analyses based on COI sequences recover a tentatively monophyletic lineage including both eastern American genera *Onomeris* Cook, 1896 and *Nearctomeris*, with a common ancestor in the Late Cretaceous to Mid Eocene and extant diversity within genera dating back to the Miocene. Our results suggest that the observed low diversity of the group in the eastern US is likely caused by extinction events, but it is also possible that new species are yet to be found. We provide new records for *Nearctomerisinexpectata* Wesener, 2012, *Onomerisunderwoodi* Cook, 1896 and *O.australora* Hoffman, 1950; the latter is here reported for the first time from South Carolina. We also present DNA barcoding data for all species of Glomerida present in the US that are not yet publicly available.

## ﻿Introduction

The order Glomerida, commonly known as pill millipedes, is a small group of Diplopoda with a mostly Holarctic distribution (Enghoff 2015). Its major center of diversity is the Western Palaearctic, especially within the Mediterranean peninsulas (Wesener 2010). In America the group is poorly diversified, and only three genera are present. The genus *Glomeroides* Chamberlin, 1922, belonging to the family Protoglomeridae, includes 15 species found in Nearctic and Neotropical forests from central Mexico to Guatemala, one present in coastal forests in Central California (Hoffman 1999), and at least one as-yet undescribed species from southern Texas (Wesener 2010).

In the eastern United States, the only known glomeridans belong to the family Glomeridae: three species in the genus *Onomeris* Cook, 1896, and a single species in the genus *Nearctomeris* Wesener, 2012 (Wesener 2010, 2012). The available distribution data of all four species is still scarce, but most records are located at low to mid elevations along the southern Appalachians. This region is one of the oldest emergent ranges in the World and a major center of diversity for several groups of organisms (e.g., Crandall and Buhay 2008; Barnes and Clark 2017), including some millipede families (Hoffman 1969; Marek and Bond 2006). The low diversity observed among pill millipedes is thus surprising, although it is possible that further undescribed species are still to be found (Hoffman 1999; Wesener 2010).

The genus *Nearctomeris* is so far known from a single species, *N.inexpectata* Wesener, 2012, only recorded from three localities in the southern Appalachian Mountains, in the states of Alabama, Tennessee and North Carolina, often in association with other pill millipede species in the genus *Onomeris* (Wesener 2012). Both genera are very similar in their general appearance and this, together with its apparently patchy and reduced distribution, may explain how *Nearctomerisinexpectata* has been overlooked until so recently. However, a closer examination of specimens shows clear differences that allows an easy diagnosis, such as the marked Y-shaped crest and antennal grooves present in *Onomeris* and missing in *Nearctomeris* (Wesener 2012). Among species, external differences are more subtle, and identification generally requires the dissection of specimens to examine the male telopods (Wesener 2010).

Here, we describe a second species of the genus *Nearctomeris* from the Great Smoky Mountains, based on morphological and molecular evidence. Also, we provide new records for *N.inexpectata*, *O.underwoodi* Cook, 1896 and *O.australora* Hoffman, 1950, and complete the DNA barcoding data for the known species of American Glomeridae. The molecular data is analyzed to propose the first hypothesis on the relationships and age of diversification within the family in North America.

## ﻿Material and methods

**Figure 1.**
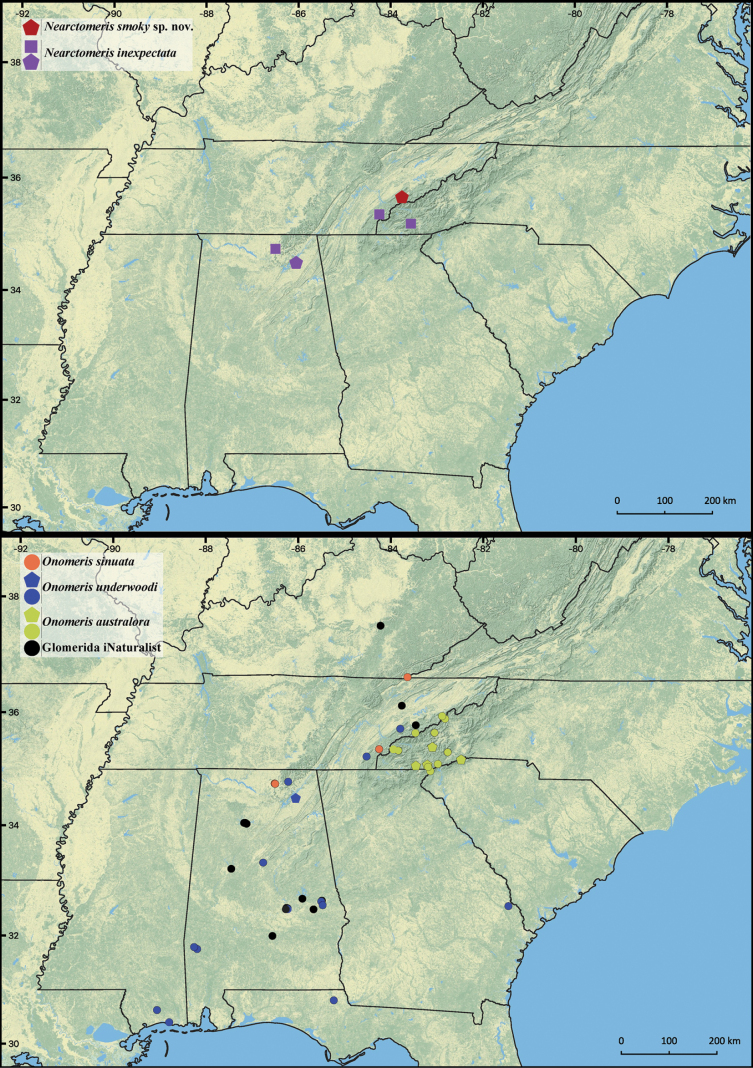
New and published records of *Nearctomeris* and *Onomeris* species, including unidentified records of eastern US Glomeridae from iNaturalist (https://www.inaturalist.org/observations?place_id=1&taxon_id=56080, accessed February 1^st^ 2023). Pentagons = new records, squares and circles = published records.

Specimens of the new species were collected by sifting leaf litter from Whiteoak Sink, near the mouth of Waterfall Cave (Tennessee: Blount Co.), in the western part of the Great Smoky Mountains National Park (Fig. 1). The litter samples were processed using Berlese-Tullgren funnels and specimens collected directly into 100% ethanol.

General habitus pictures were generated by focus stacking 20 images taken with a Nikon EOS 6D camera equipped with a Tamron AF 1.4× teleconverter and a Canon MP-E 65 mm macro lens, using a Visionary Digital Passport system, and combined with Helicon Focus software v.8.1.1 (HeliconSoft, Ukraine). Morphological examination was performed using an Olympus SZX7 stereomicroscope, and a Zeiss Axioskop 50 compound microscope. Drawings were prepared using a camera lucida and digitized using a Wacom Intuos Pro tablet. Tegument details were examined using a Hitachi S3400N variable pressure scanning electron microscope (SEM), using uncoated specimens.

We studied additional material (Table 1, Suppl. material 1) corresponding to other species of Glomeridae from eastern US, including samples collected by the authors and colleagues, and others found among sample residues (i.e., leftovers of former samplings after pulling out specimens of immediate interest) stored in the Clemson University Arthropod Collection and made readily searchable online by posting photos of the different samples (https://www.cuacinsects.org/databases.html). We also collected records of eastern US Glomeridae from published papers (Loomis 1943; Hoffman 1950; Causey 1959; Shelley 2000; Wesener 2010, 2012) and the citizen science initiative iNaturalist (https://www.inaturalist.org) (Suppl. material 1). Maps showing these records were generated using QGIS v.3.30 (http://qgis.org/). The type material of the new species is deposited at the U.S. National Entomological Collection (**USNM**, Smithsonian National Museum of Natural History), the collections of the Great Smoky Mountains National Park (**GRSM**) and the Clemson University Arthropod Collection (**CUAC**) (see examined material). All non-type material is deposited at CUAC.

**Table 1.** Extracted and sequenced specimens of *Nearctomeris* Wesener, 2012 and *Onomeris* Cook, 1896, and GenBank sequences included in the phylogenetic analyses.

Species	Voucher	Locality	GPS coordinates	Elevation (m)	Collection date	GenBank Acces. #	Reference	Comments
*Nearctomerissmoky* sp. nov.	GRSM217979	USA: TN: Blount Co.: Smoky Mountains N. P.: White Oak Sink	35.6369°N, 83.7418°W	357	27.x.2021	OQ833533	This study	-
*Nearctomerissmoky* sp. nov.	GRSM217980	USA: TN: Blount Co.: Smoky Mountains N. P.: White Oak Sink	35.6369°N, 83.7418°W	357	27.x.2021	OQ833534	This study	-
*Nearctomerissmoky* sp. nov.	CUAC000180808	USA: TN: Blount Co.: Smoky Mountains N. P.: White Oak Sink	35.6369°N, 83.7418°W	357	27.x.2021	OQ833535	This study	-
*Nearctomerissmoky* sp. nov.	CUAC000180810	USA: TN: Blount Co.: Smoky Mountains N. P.: White Oak Sink	35.6369°N, 83.7418°W	357	27.x.2021	OQ833536	This study	-
*Nearctomerisinexpectata*	CUAC000180813	USA: AL:Dekalb Co.: Grove Oak: Buck’s Pocket	34.4716°N, 86.0523°W	296	5.iv.2022	NUMT	This study	New record
*Nearctomerisinexpectata*	CUAC000180814	USA: AL:Dekalb Co.: Grove Oak: Buck’s Pocket	34.4716°N, 86.0523°W	296	5.iv.2022	NUMT	This study	
*Nearctomerisinexpectata*	ZFMK-MYR005	USA: AL: Madison Co.: Huntsville: Monte Sano State Park	34.7362°N, 86.5000°W	324	30.iii.–01.iv.2010	JQ074185	Wesener 2012	-
*Nearctomerisinexpectata*	ZFMK-MYR008	USA: AL: Madison Co.: Huntsville: Monte Sano State Park	34.7362°N, 86.5000°W	324	30.iii.–01.iv.2010	JQ074186	Wesener 2012	-
*Onomerisunderwoodi*	CUAC000180816	USA: AL:Dekalb Co.: Grove Oak: Buck’s Pocket	34.4716°N, 86.0523°W	296	5.iv.2022	OQ833539	This study	New record
*Onomerisunderwoodi*	CUAC000180817	USA: AL:Dekalb Co.: Grove Oak: Buck’s Pocket	34.4716°N, 86.0523°W	296	5.iv.2022	OQ833540	This study	
*Onomerisaustralora*	CUAC000180819	USA: NC: Macon Co.: Highlands: Highlands Biological Station	35.0530°N, 83.1880°W	1206	18.viii.2016	OQ833537	This study	-
*Onomerisaustralora*	CUAC000180820	USA: NC: Macon Co.: Highlands: Highlands Biological Station	35.0530°N, 83.1880°W	1206	18.viii.2016	OQ833541	This study	-
*Onomerisaustralora*	CUAC000180821	USA: NC: Jackson Co.: Balsam Mountain Preserve	35.3751°N, 83.1025°W	1512	20.vii.2015	OQ833538	This study	New record
*Onomerisaustralora*	CUAC000180822	USA: SC: Pickens Co.: Eastatoe Creek Heritage Pres.	35.1577°N, 82.4910°W	678	30.ii.2015	NUMT	This study	First record for South Carolina
*Onomerisaustralora*	CUAC000180823	USA: SC: Pickens Co.: Eastatoe Creek Heritage Pres.	35.1577°N, 82.4910°W	678	30.ii.2015	OQ833542	This study	
*Onomerisaustralora*	CUAC000180825	USA: NC: Macon Co.: Otto: Coweeta Hydrologic Laboratory	35.0288°N, 83.4594°W	1450	13.xii.2022	NUMT	This study	New record
*Onomerisaustralora*	CUAC000180826	USA: NC: Macon Co.: Otto: Coweeta Hydrologic Laboratory	35.0288°N, 83.4594°W	1450	13.xii.2022	NUMT	This study	
*Onomerissinuata*	FMNH-INS-56316	USA: AL: Madison Co.: Huntsville: Monte Sano State Park	34.7362°N, 86.5000°W	324	30.iii.–01.iv.2010	JQ074183	Wesener 2012	-
*Onomerissinuata*	FMNH-INS-56316	USA: AL: Madison Co.: Huntsville: Monte Sano State Park	34.7362°N, 86.5000°W	324	30.iii.–01.iv.2010	JQ074184	Wesener 2012	-
*Glomeroidesprimus*	ZFMK-MYR004	USA: CA: Mendocino Co.: Jackson Demonstration State Forest	39.3976°N, 123.6946°W	35	29.iii.2011	JQ074182	Wesener 2012	-
*Trachysphaeralobata*	ZFMK-MYR-924	France: Aquitaine: Dép. Dordogne: Génis, Gorges de l’Auvézère	-	-	-.viii.2011	KJ408482	Wilbrandt et al 2015	-
*Protoglomerisvasconica*	ZFMK-MYR-934	Spain: Galicia: Lugo: Trabada	43.4295°N, 7.2290°W	393	29.vii.2012	KP205572	Oeyen and Wesener 2015	-
*Trachysphaeraschmidtii*	ZFMK-MYR-BGIMyr16	Croatia: Primorje-Gorski Kotar: Rijeka	-	-	15.x.2011	KJ408481	Wilbrandt et al 2015	-
*Glomeridellaminima*	BC ZSM MYR 00199	Austria: Oberoesterreich: Kaltenbach, NNE Ruine Wildenstein	47.702°N, 13.604°E	510	3.iv.2010	HQ966139	Spelda et al. 2011	-
*Hyleoglomerishalang*	IEBR_Myr_926	Vietnam: Cao Bang Prov.: Ha Lang Dist.: Duc Quang, Nguom Hang cave	22.7208°N, 106.6692°E	516	15.iii.2022	ON704754	Kuroda et al. 2022a	-
*Hyleoglomerisawaumi*	EG20210711-227-01	Japan: Shiga Pref., Omihachiman-shi,Miyauchi-cho, Hachiman-koen	35.1422°N, 136.0850°E	152	11.vii.2021	LC713407	Kuroda et al. 2022b	-
*Hyleoglomerisinsularum*	EG20201213-09	Japan: Kanagawa Pref., Odawara-shi, Nebukawa	-	-	13.xii.2020	LC713421	Kuroda et al. 2022b	-
*Hyleoglomerisjaponica*	MS20210617-01	Japan: Kanagawa Pref., Fujisa-wa-shi, Enoshima Island	-	-	17.vi.2021	LC713422	Kuroda et al. 2022b	-
*Hyleoglomerislucida*	EG20210718-240-01	Japan: Saitama Pref., Chichi-bu-shi, Kamikagemori	-	-	18.vii.2021	LC713425	Kuroda et al. 2022b	-
*Hyleoglomerissulcata*	MS20210521B-05	Japan: Kanagawa Pref., Zushi-shi,Numata, Jimmuji	-	-	21.v.2021	LC713428	Kuroda et al. 2022b	-
*Hyleoglomerisuenoi*	ST20211028	Japan: Yamaguchi Pref., Ube-shi,Higashikibe	-	-	28.x82021	LC713429	Kuroda et al. 2022b	-
*Hyleoglomerislobus*	SVE-204	Vietnam	-	-	-	MT749391	Nguyen et al. 2021	-
*Hyleoglomeris* sp.	IEBR-721	Vietnam	-	-	-	MT749399	Nguyen et al. 2021	-
*Hyleoglomeris* sp.	IEBR-834	Vietnam	-	-	-	MT749393	Nguyen et al. 2021	-
*Hyleoglomeris* sp.	IEBR-823	Vietnam	-	-	-	MT749395	Nguyen et al. 2021	-
*Tonkinomerishuzhengkuni*	SCAU TY01	China: Guizhou Prov.: Tongren City: Jiangkou County: Baishuidong Scenic Area	27.6529°N, 108.7952°E	450	25.xi.2019	MT522013	Liu and Golovatch 2020	-
*Tonkinomerisnapoensis*	IEBR-804b	Vietnam	-	-	-	MT749396	Nguyen et al. 2021	-
*Rhopalomerissauda*	IEBR-533	Vietnam	-	-	-	MT749404	Nguyen et al. 2021	-
*Rhopalomerisnagao*	IEBR-852	Vietnam: Cao Bang Province: Pia Oac – Pia den National Park	22.6082°N, 105.8693°E	1600	7.vi.2020	MT749392	Nguyen et al. 2021	-
*Peplomerismagna*	IEBR-677	Vietnam	-	-	-	MT749405	Nguyen et al. 2021	-
*Hyperglomerissimplex*	IEBR-605	Vietnam	-	-	-	MT749403	Nguyen et al. 2021	-
*Hyperglomeris* sp.	IEBR-674	Vietnam	-	-	-	MT749409	Nguyen et al. 2021	-
*Onychoglomeristyrolensis*	ZFMK-MYR-1276	Italy: Trentino-Alto Adige: Trento	-	-	-.v-2012	KP205571	Oeyen and Wesener 2015	-
*Glomerellinalaurae*	ZFMK-MYR-2260	Greece: South Aegean: Rhodes: Kapi	-	-	1.i.2000	KP205573	Oeyen and Wesener 2015	-
*Eupeyerimhoffiaarchimedis*	ZFMK-MYR-1876	Italy: Sicily: Syracuse:Ferla	37.1151°N, 014.9404°E	452	10.vii.2013	KP205574	Oeyen and Wesener 2015	-
*Simplomerismontivaga*	ZFMK-MYR-2622	Switzerland: Valais: Riederalp	46.3825°N, 8.0223°E	-	22.vi.2014	OP602221	Wesener 2022	-
*Haploglomerismultistriata*	ZFMK-MYR-1354	Austria: Niederösterreich: Puchberg am Schneeberg	47.7894°N ,15.8152°E	1240	14.iv.2011	OP602220	Wesener 2022	-
*Glomerismaerens*	ZFMK-TIS-2517208	Spain: Barcelona: Castellet, El Vendrell	-	-	-	MG892111	Reip and Wesener 2018	-
*Glomerisklugii*	BC ZSM MYR 00192	Germany: Bavaria: Langenaltheimer Haardt	48.890°N, 10.979°E	562	13.vi.2010	HQ966135	Spelda et al. 2011	-
*Glomerismarginata*	BC ZSM MYR 00045	Germany: Rhineland Palatinate: Rheinbreitbach	50.619°N, 7.254°E	181	1.x.2009	HM888107	Spelda et al. 2011	-
*Glomerisconnexa*	BC ZSM MYR 00025	Germany: Bavaria: 1 km WSW Scheidegg	47.577°N, 9.835°E	812	21.x.2009	HM888094	Spelda et al. 2011	-
*Glomerispustulata*	BC ZSM MYR 00022	Germany: Bavaria: Veste Oberhaus	48.578°N, 13.468°E	389	11.x.2009	HM888091	Spelda et al. 2011	-
*Glomeristetrasticha*	BC ZSM MYR 00033	Germany: Bavaria: Partnachklamm	47.475°N, 11.115°E	751	30.ix.2009	HM888102	Spelda et al. 2011	-
*Glomerisintermedia*	BC ZSM MYR 00029	Germany: Rhineland Palatinate: Rheinbreitbach	50.619°N, 7.254°E	181	1.x.2009	HM888098	Spelda et al. 2011	-
*Glomerisornata*	BC ZSM MYR 00021	Germany: Baden-Wuerttemberg: Unterwilzingen	48.260°N, 9.536°E	613	05.xi.2009	HM888090	Spelda et al. 2011	-
*Glomerisapuana*	ZFMK-MYR-753	Italy: Liguria: Cinque Terre	44.1261°N, 9.7258°E	510	25.ix.2009	KT188944	Wesener 2015a	-
*Glomerisligurica*	ZFMK-MYR-4256	Italy: Liguria: 2 km NW Campo Ligure	44.5441°N, 8.6837°E	338	15.iv.2011	KT188950	Wesener 2015a	-
*Glomerishelvetica*	ZFMK-MYR-4290	Switzerland: Valais: Sion	46.23°N, 7.35°E	493	22.iv.2015	KR997499	Wesener 2015b	-
*Glomerisvalesiaca*	ZFMK-MYR-829	Switzerland: Valais: Sion	46.23°N, 7.35°E	493	-	KR997494	Wesener 2015b	-
*Glomeristranslapina*	ZFMK-MYR-2636	Switzerland: Valais: Simplonpass	46.2473°N, 8.0388°E	2130	23.vi.2014	KX714039	Wesener and Conrad 2016	-
*Glomerisprimordialis*	ZFMK-MYR-4745	Italy: Piemonte: Biella: Pollone—Favaro	45.5894°N, 8.003°E	626	13.iv.2011	KX714048	Wesener and Conrad 2016	-
*Glomerisoblongoguttata*	ZFMK-MYR-4568	Italy: Lombardia: Brescia: Pisogne	45.7985°N, 10.1152°E	281	9.iv.2011	KX714045	Wesener and Conrad 2016	-
*Glomerisoropensis*	ZFMK-MYR-4534	Italy: Piemonte: Biella: NW Sanctuary of Oropa	45.6295°N, 7.9817°E	1200	14.iv.2011	KX714040	Wesener and Conrad 2016	-
*Glomerisromana*	ZFMK-MYR-797	San Marino: Città di San Marino Wiese unter Steine	-	-	16.ix.2009	KX714036	Wesener and Conrad 2016	-

We extracted DNA from several specimens (Table 1), including the new species, using the GeneJET Genomic DNA Purification Kit (Thermo Fisher Scientific, Waltham, MA, USA), following standard protocol, and eluting in molecular grade water. We amplified a 658 bp fragment of the barcoding region of the Cytochrome Oxidase Subunit I (COI) mitochondrial gene using the primers LCO1490 and HCO2198 (Folmer et al. 1994). Polymerase chain reaction (PCR) reactions were performed in a 25 μL volume using the conditions described in Recuero and Rodríguez-Flores (2020). We visualized PCR products in a 1% agarose gel electrophoresis to check PCR success, and sent them to Psomagen, Inc. (Maryland, USA) for cleaning and sequencing. Sequences are deposited in GenBank (Table 1).

Sequences were compiled, assembled, and edited using Sequencher v.5.4.1 (Gene Codes Corporation), and aligned manually, including several Glomerida taxa available in GenBank from previous works (Spelda et al. 2011; Wesener 2012, 2015a, 2015b, 2022; Oeyen and Wesener 2015; Wilbrandt et al 2015; Wesener and Conrad 2016; Reip and Wesener 2018; Liu and Golovatch 2020; Nguyen et al. 2021; Kuroda et al. 2022a, 2022b) (Table 1). Sequences were translated to amino acids to check the presence of stop codons; this way, we were able to identify a few of our fragments as nuclear mitochondrial DNA (NUMT), which were removed from our analyses (Table 1). Mean uncorrected pairwise genetic distances (p-distances) among species were calculated using MEGA6 (Tamura et al. 2013). Phylogenetic hypotheses were generated on an unpartitioned matrix using maximum likelihood (ML) and a Bayesian approach. ML analyses were performed with W-IQ-TREE (available at http://iqtree.cibiv.univie.ac.at; Trifinopoulos et al. 2016), allowing the program to select the best fitting substitution model, and measuring branch support with 1000 ultrafast bootstrap replicates. Bayesian phylogenetic inference, including estimates of time to the most recent common ancestor (TMRCA), was performed with BEAST v.1.10.4 (Drummond et al. 2012); we used a GTR+G+I substitution model as estimated in the previous analysis, a birth–death tree prior and a lognormal relaxed molecular clock; given our limited taxonomic sampling and data, the few available fossil records for Glomerida could not be adequately incorporated as a calibration point into the analysis (Wesener 2019), so we fixed a substitution rate of 2.3%/Myr (0.0115 substitution/site/Myr), widely used for ArthropodaCOI sequence data (Brower 1994). The analysis was run for 100 million generations, sampling every 10000, and repeated independently four times to check consistency of the results, yielding in all cases high effective sample sizes (> 200) for all parameters as checked with Tracer v.1.7.2 (Rambaut et al. 2018). A maximum clade credibility tree was built with TreeAnnotator v.1.10.4 considering a 25% burn-in.

## ﻿Results

**Figure 2.**
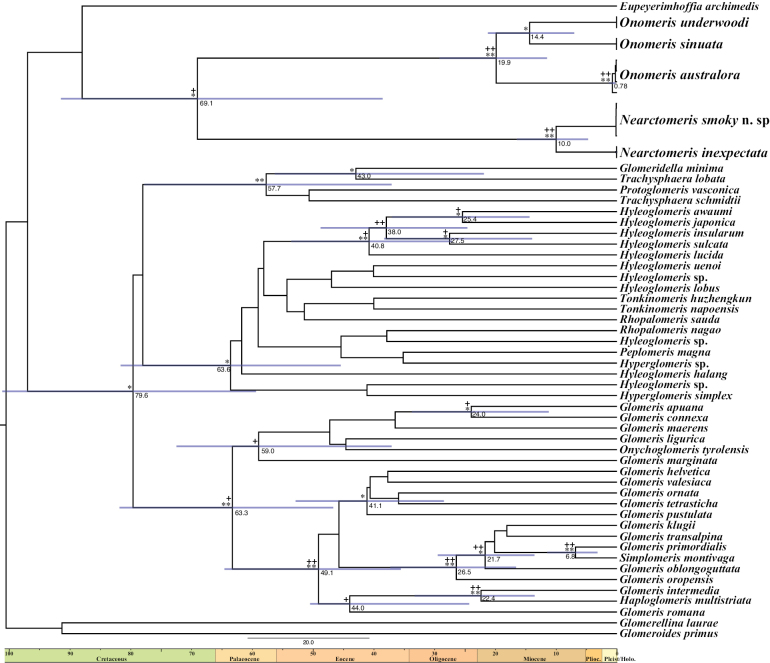
Bayesian chronogram based on COI sequences. Support is indicated by the nodes: ** = BEAST posterior probabilities > 0.95, * = BEAST posterior probabilities > 0.80 < 0.95, ++ = ML ultrafast bootstrap > 95, + = ML ultrafast bootstrap > 0.80 < 0.95. Values by supported nodes are the mean values of TMRCA; blue bars on nodes reflect TMRCA's 95% HDP.

The phylogenetic analyses results are limited by using just a 658 bp fragment of the mitochondrial COI gene, and most clades lack support in either ML or BEAST trees, especially in the basal relationships (Fig. 2). As expected, we observe a sister relationship between *Nearctomerisinexpectata* and *N.smoky* sp. nov., with a mean genetic p-distance of 6.85%. The clade including all *Onomeris* species is also well supported, with *O.australora* being the sister lineage to a clade containing *O.sinuata* (Loomis, 1943) and *O.underwoodi*, although again these relationships are weakly supported, especially in the ML analysis. Mean genetic p-distances are 7.58% between *O.sinuata*-*O.underwoodi*, 10.82% between *O.sinuata*-*O.australora*, and 11.32% between *O.underwoodi*-*O.australora*. Although with only moderate support, we recover a monophyletic clade including both *Onomeris* and *Nearctomeris*. This American Glomeridae clade dates back to the Late Cretaceous to Mid Eocene, while the ages of *Onomeris* and *Nearctomeris* species are estimated around the Mid and Late Miocene, respectively (Fig. 2).

New records of *Nearctomerisinexpectata*, *Onomerisunderwoodi* and *Onomerisaustralora* are provided in Table 1 and shown in Fig. 1. *Onomerisaustralora* is reported here for the first time in the state of South Carolina, within the range of the Blue Ridge Mountains.

### ﻿Taxonomy

**Order Glomerida Brandt, 1833**

**Family Glomeridae Leach, 1815**

**Genus *Nearctomeris* Wesener, 2012**

#### ﻿ Nearctomeris smoky sp. nov.

Taxon classificationAnimaliaGlomeridaGlomeridae

3213F12F-C533-559E-9836-A3EACCB4D215

https://zoobank.org/ECD780D7-C94D-46C4-9C35-D517EA974758

Figs 3 , 4 , 5

##### Type material.

**Figure 3.**
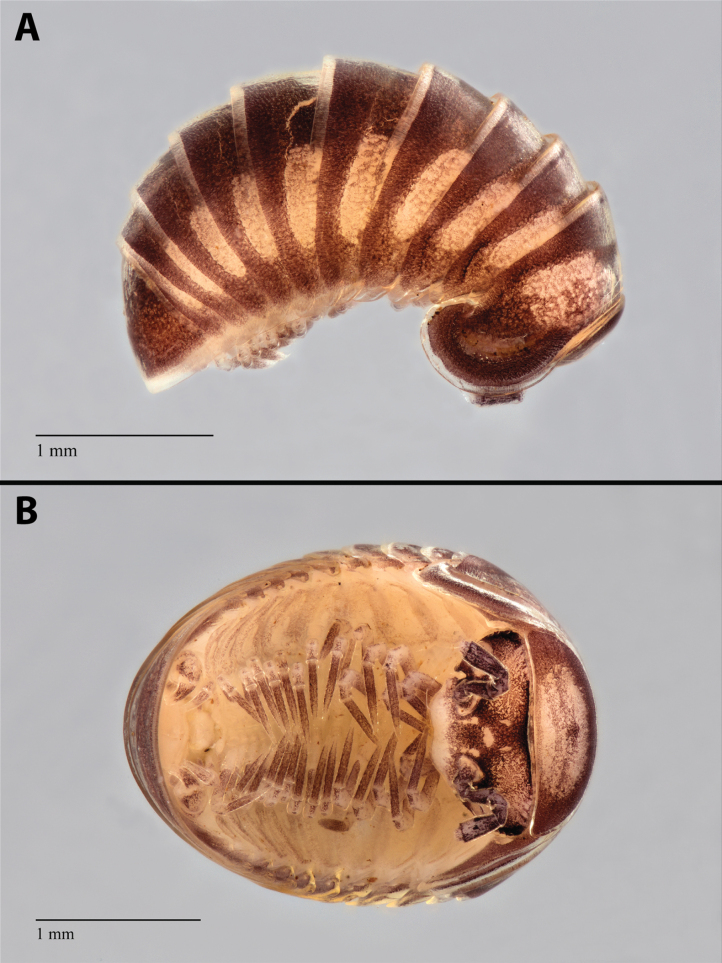
*Nearctomerissmoky* sp. nov., habitus of male holotype (USNM) **A** lateral view **B** ventral view.

***Holotype***: male (USNM ENT01838998; Fig. 3), USA, Tennessee, Blount Co., Great Smoky Mountains N. P., Whiteoak Sink; 35.6369°N, 83.7418°W; in leaf litter at base of rock; leg. M. Caterino, A. Haberski & P. Wooden, 27.x.2021. ***Paratypes***: 2 males (CUAC000180803, GRSM217979), 2 females (USNM ENT01838999, GRSM217980) and 5 juveniles (CUAC000180807–CUAC000180810, GRSM217981), same data as holotype.

##### Diagnosis.

Externally similar to *Nearctomerisinexpectata*; they can be differentiated in the shape of the femoral process (distal finger) of telopods (Fig. 5E), blunt and rounded in *N.inexpectata*, elongated and curved in the *N.smoky* sp. nov., and of the syncoxite (Fig. 5D), distally broader in *N.inexpectata* than in *N.smoky* sp. nov., with rounded central lobe in *N.inexpectata* and bilobed in *N.smoky* sp. nov., and lateral processes shorter in *N.inexpectata* than in *N.smoky* sp. nov. The observed mean COI pairwise uncorrected p-distance between both species is 6.85%.

##### Name.

Smoky, a noun in apposition, refers to the Great Smoky Mountains where the species lives.

##### Description.

Body with 12 segments (including collum). Length of largest male (holotype), 2.9 mm; width at thoracic shield 1.8 mm, at tergite five 2 mm; height of thoracic shield 1.3 mm. Length of largest female 3.2 mm; width at thoracic shield 2 mm, at tergite five 2.1 mm; height of thoracic shield 1.3 mm.

**Figure 4.**
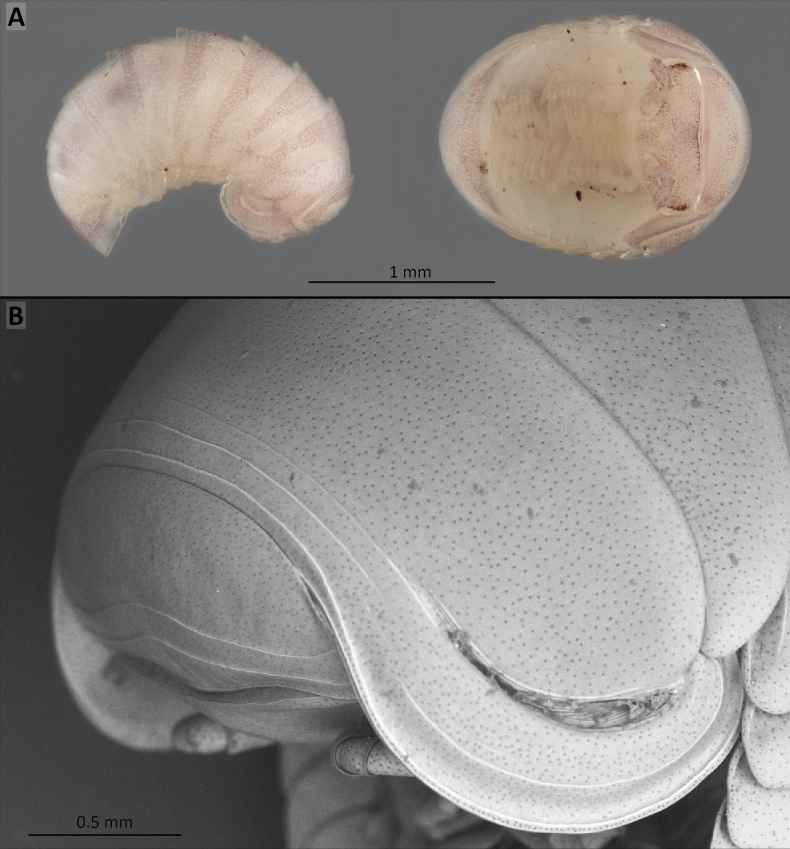
*Nearctomerissmoky* sp. nov. **A** habitus of a 10-segment juvenile (CUAC) in lateral and ventral views **B**SEM image of collum and thoracic shield of a female (USNM), showing details of tegument, striae and schism.

General coloration of adults (Fig. 3) brown to dark brown dorsally; collum with a large, central, off-white area; tergites 2–11 with lateral, transversely oval, off-white areas; lateral and posterior margins of tergites translucent; head brown, more or less mottled with white, labrum and organ of Tömösváry white, ocular field black; antennae brown mottled with white; ventrally off-white, legs white, mottled or not with brown, with brown tarsi. Juveniles with 11 segments with similar pattern but much lighter than adults, and even lighter in juveniles with 10 segments (Fig. 4A).

***Head*** (Fig. 3B): structure typical of *Nearctomeris*, without any distinct crest or groove. Ommatidia 5+1 or 4+1, unpigmented, within a black, elongate ocular field. Tömösváry’s organ transverse, horseshoe-shaped, about 2 times as wide as long. Antennae with antennomere 3 shorter than 1 and 2 combined; four apical cones.

***Collum*** (Fig. 4B): surface smooth, finely and densely punctured dorsally with minute pits, as the rest of segments, with two well-marked, transverse striae.

***Thoracic shield*** (Fig. 4B): with schisms rounded posteriorly, well-differentiated but not protruding beyond tergite contour. Schism impression broad and well-developed. Only three striae transversely crossing the shield; no trace of a central, incomplete stria. One strong, one very weak lateral stria below the schism impression.

***Tergites*** (Figs 3A, 4A): with soft, shiny appearance; surface densely covered with small pits that, observed through the transparent first layer of the tegument, seem to be the opening of pore canals connecting the epidermis with the exterior. There is no trace of setae on the tergites. Tergite 11 is partially hidden under tergite 10.

**Figure 5.**
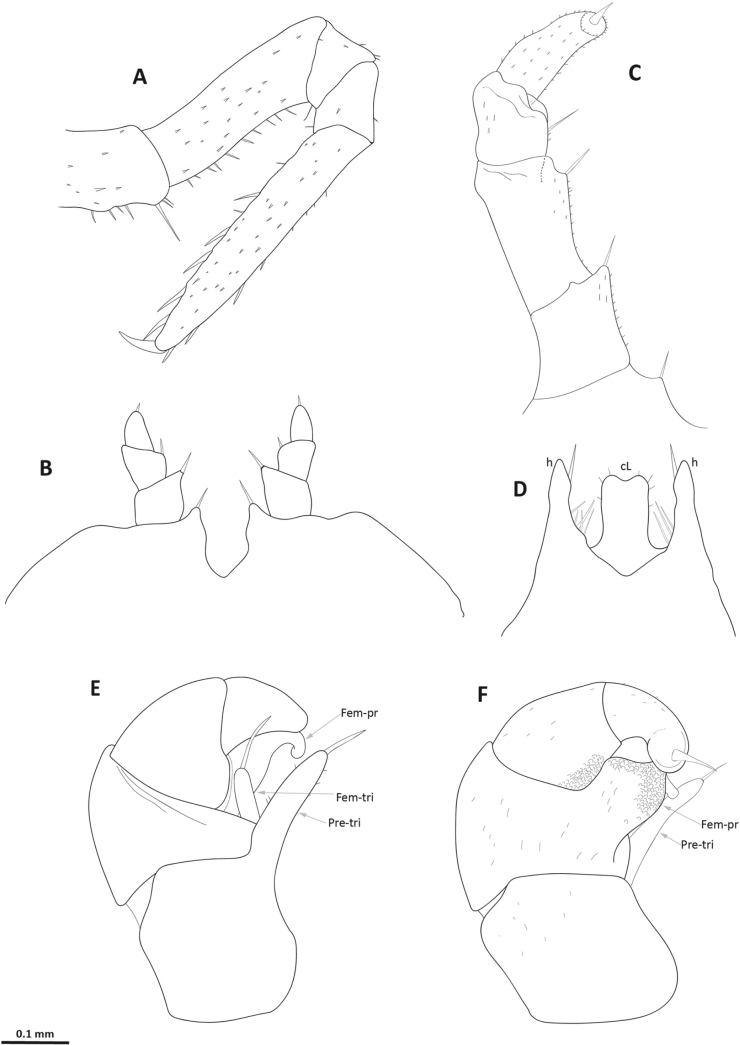
*Nearctomerissmoky* sp. nov., holotype (USNM) **A** male left leg 11, anterior view **B** male leg pair 17, anterior view **C** male left leg 18, anterior view **D** telopod syncoxite, anterior view **E** left telopod, anterior view **F** left telopod, posterior view. Abbreviations: **cL** = syncoxite central lobe; **Fem-pr** = telopod femoral process; **Fem-tri** = telopod femoral trichostele; **h** = syncoxite lateral process; **Pre-tri** = telopod prefemoral trichostele.

***Mid-body legs*** (Fig. 5A): relatively slender, femur about 2.5 times as long as wide, tarsus 5–5.5 times as long as wide. Ventral margin of prefemur and femur with numerous strong setae. Tarsus with 5–6 ventral, 2–3 dorsal spiniform setae, mostly set in the distal half. Claw 4–4.5 times as long as wide.

***Anal shield*** (Fig. 3B): with evenly rounded posterior border, with no sign of a notch or concavity.

***Male leg pair 17*** (Fig. 5B): with a broad coxal lobe, mesally with a spiniform seta; telopodite formed by three strongly reduced podomeres, first and second with mesal spiniform setae, third with an apical spiniform seta.

***Male leg pair 18*** (Fig. 5C): with no obvious syncoxial notch; coxa with a spiniform seta; 4-segmented telopodite, reduced but better developed than in leg pair 17. First, second and third podomeres with mesal spiniform setae, apical in fourth podomere.

***Telopod*** (Fig. 5D–F): robustly developed; syncoxite (Fig. 5D) with a long, subrectangular central lobe (cL) with bilobed distal margin, flanking lateral processes (h) longer than the lobe and carrying long, strong, mesoproximal setae and a spiniform mesodistal seta. Prefemur trichostele (Fig. 5E, F; **Pre-tri**) about as long as prefemur width. Femur trichostele (Fig. 5E; **Fem-tri**) about two thirds as long as the prefemoral one. Femoral process (distal finger) (Fig. 5E, F; **Fem-pr**) broad at base, distal half much narrower and distal third curved anteriad. Fields of sclerotized scale-like structures present on femoral distal finger and tibia (Fig. 5F). Tarsus strong, blunt and curved, with a strong, apical spine.

## ﻿Discussion

Some of the oldest fossils of Oniscomorpha, a probably non-monophyletic group including the different orders of pill millipedes (Benavides et al. 2023), have been found at the Middle Pennsylvanian deposits of Mazon Creek, Illinois, US, with an age of over 300 Myr (Hannibal and Feldmann 1981). Although most of the species belong to the already extinct order Amynilyspedida, some fossils were tentatively assigned to Sphaeroteriida and, according to some authors, they could even correspond to Glomerida (Shelley and Golovatch 2011, but see Racheboeuf et al. 2004). However, the current diversity of oniscomorph millipedes in America is notably impoverished, with only a few known species of Glomerida (Hoffman 1999).

In the eastern United States, with only five species in two genera, the low known diversity of pill millipedes could be explained by different reasons. On one hand it could be caused by a relatively recent colonization, with little time for subsequent diversification. It has been hypothesized that both genera might not represent sister lineages and that they could be more closely related to Asian taxa such as *Hyleoglomeris* Verhoeff, 1910 and *Hyperglomeris* Silvestri, 1917 rather than to each other (Wesener 2012, but see Liu and Golovatch 2020). This hypothesis is compatible with the recent colonization scenario, but also with old events of dispersal and extinction on intervening geographic areas, as has been proposed for some species in the millipede genus *Brachycybe* Wood, 1864 (Brewer et al. 2012).

Alternatively, the current species could represent relics of a formerly more diverse fauna affected by high extinction rates, in which case we would expect a sister relationship between *Onomeris* and *Nearctomeris*. Our phylogenetic analyses favor this idea, supporting a monophyletic lineage including both genera, with a common ancestor dating back most likely to the Mid Eocene, and no close relationship with any of the Palaearctic taxa included in the analyses. However, the proposed relationships are tentative, being based in a single locus, likely affected by saturation, and suffering from reduced taxonomic sampling considering the global diversity of Glomerida, as has happened in previous attempts to resolve the phylogeny of the order using molecular data (Oeyen and Wesener 2015; Liu and Golovatch 2020). Diversification of extant lineages within *Onomeris* and *Nearctomeris* occurred during the Middle and Upper Miocene, resulting in long branches that are suggestive of diversity loss, and supporting the hypothesis of old relict species of a more diverse fauna affected by high extinction rates. The clades including the Asian *Hyleoglomeris*, or the European *Glomeris* Latreille, 1802, exhibit long but densely bifurcated branches, suggesting that extinction has been considerably lower in those lineages than in eastern US taxa.

Our age estimates must be considered as tentative, considering the limitation of our dataset and that they are based on a substitution rate that, even if widely used across different groups of Arthropoda including millipedes (e.g., Brewer et al. 2012; Nielsen et al. 2022), could be different in a group like Glomerida. It has been found that inter- and intraspecific distances in other glomeridan genera, such as *Glomeris*, are unusually high for COI sequences (Wesener 2015a; Wilbrandt et al. 2015; Wesener and Conrad 2016; Reip and Wesener 2018). This could indicate either a faster substitution rate or, alternatively, old speciation events or presence of cryptic species not yet delimited; however, we still do not have the necessary data to test these scenarios. In the case of a faster substitution rate, our estimates would be overly old, and the actual ages of the clades would be more recent.

However, we must not discount the likelihood that there is still some diversity that has not been identified yet. In fact, the strong morphological conservatism typical of both genera could be hiding a higher diversity than reflected by current taxonomy, and it has been generally assumed that further species should be found and described (Hoffman 1999; Shelley 2000). An integrative taxonomic approach could help detect those cases, both in *Onomeris* and *Nearctomeris*, as has been shown in other Appalachian millipedes, both helping describe new species (e.g., Means et al. 2021) or synonymizing taxa (e.g., Vasquez-Valverde and Marek 2022).

In the case of *Onomerisunderwoodi* a moderate interspecific morphological variability has been described (Causey 1959; Wesener 2010); considering its wide distribution it could be possible that there are morphologically similar or even cryptic species under that name. Unfortunately, we still lack sufficient sampling to tackle this point. The best represented species in our analyses, *Onomerisaustralora*, has shown shallow genetic divergences in the southern portion of its distribution, but we have no data from populations further north. *Onomerissinuata* is known from just a few, isolated and widely separated localities; it could be that the species has not been found yet in intervening areas, but if the isolation is real then the existence of independent lineages or even species may be predicted; a similar pattern is observed within *Nearctomerisinexpectata* (Wesener 2012). Glomerids are widespread in the eastern US, ranging from Florida and Mississippi in the south to Kentucky in the north, but within that huge region there are large areas where no reports are available. Further sampling, together with detailed morphological and molecular characterization are still necessary to determine the real diversity of pill millipedes in the eastern US.

## Supplementary Material

XML Treatment for Nearctomeris smoky

## ﻿Additional information

### Author ORCIDs

Ernesto Recuero https://orcid.org/0000-0001-8086-8667

Michael S. Caterino https://orcid.org/0000-0002-2597-5707

### Data availability

All of the data that support the findings of this study are available in the main text or Supplementary Information.

## Supplementary materials

Supplementary material 1 Records of Glomeridae in the eastern United States Data type Localities (text file) Explanation note Published and new records of Glomeridae in the eastern United States. https://binary.pensoft.net/file/863260This dataset is made available under the Open Database License (http://opendatacommons.org/licenses/odbl/1.0/). The Open Database License (ODbL) is a license agreement intended to allow users to freely share, modify, and use this Dataset while maintaining this same freedom for others, provided that the original source and author(s) are credited.Ernesto Recuero, Michael S. Caterino

## References

[B1] Barnes R Clark AT (2017) Sixty-five million years of change in temperature and topography explain evolutionary history in Eastern North American plethodontid salamanders. American Naturalist 190(1): E1–E12. 10.1086/691796 28617631

[B2] Benavides LR Edgecombe GD Giribet G (2023) Re-evaluating and dating myriapod diversification with phylotranscriptomics under a regime of dense taxon sampling. Molecular Phylogenetics and Evolution 178: 107621. 10.1016/j.ympev.2022.107621 36116731

[B3] Brandt JF (1833) Tentaminum quorundam monographicorum InsectaMyriapoda Chilognathi Latreillii spectantium prodromus. Bulletin de la Societé Impériale des Naturalistes de Moscou 6: 194–209.

[B4] Brewer MS Spruill CL Rao NS Bond JE (2012) Phylogenetics of the millipede genus *Brachycybe* Wood, 1864 (Diplopoda: Platydesmida: Andrognathidae): Patterns of deep evolutionary history and recent speciation. Molecular Phylogenetics and Evolution 64(1): 232–242. 10.1016/j.ympev.2012.04.003 22516430

[B5] Brower AVZ (1994) Rapid morphological radiation and convergence among races of the butterfly *Heliconiuserato* inferred from patterns of mitochondrial DNA evolution. Proceedings of the National Academy of Sciences of the United States of America 91(14): 6491–6495. 10.1073/pnas.91.14.6491 8022810 PMC44228

[B6] Causey NB (1959) New records of *Onomerisunderwoodi* Cook (Diplopoda: Glomerida: Glomeridae). Proceedings of the Biological Society of Washington 72: 151–154.

[B7] Chamberlin RV (1922) The millipeds of Central America. Proceedings of the United States National Museum 60: 1–75. 10.5479/si.00963801.60-2403.1

[B8] Cook OF (1896) An American Glomeroid. Brandtia 10: 43–45.

[B9] Crandall KA Buhay JE (2008) Global diversity of crayfish (Astacidae, Cambaridae, and Parastacidae—Decapoda) in freshwater. Hydrobiologia 595(1): 295–301. 10.1007/s10750-007-9120-3

[B10] Drummond AJ Suchard MA Xie D Rambaut A (2012) Bayesian phylogenetics with BEAUti and the BEAST 1.7. Molecular Biology and Evolution 29(8): 1969–1973. 10.1093/molbev/mss075 22367748 PMC3408070

[B11] Enghoff H (2015) Diplopoda—geographical distribution. In: Minelli A (Ed.) Treatise on Zoology – anatomy, taxonomy, biology.The Myriapoda, vol. 2. Brill, Leiden, 329–326. 10.1163/9789004188273_014

[B12] Folmer O Black M Hoeh W Lutz R Vrijenhoek R (1994) DNA primers for amplification of mitochondrial cytochrome c oxidase subunit I from diverse metazoan invertebrates. Molecular Marine Biology and Biotechnology 3: 294–299. 7881515

[B13] Hannibal JT Feldmann RM (1981) Systematics and functional morphology of oniscomorph millipedes (Arthropoda: Diplopoda) from the Carboniferous of North America. Journal of Paleontology 55: 730–746.

[B14] Hoffman RL (1950) Records and descriptions of diplopods from the southern Appalachians. Journal of the Elisha Mitchell Scientific Society 66: 11–33.

[B15] Hoffman RL (1969) The origin and affinities of the southern Appalachian diplopod fauna. In: Holt PC (Ed.) The Distributional History of the Biota of the Southern Appalachians Part 1: Invertebrates.Research Division Monograph 1, Virginia Polytechnic Institute, Blacksburg, 221–246.

[B16] Hoffman RL (1999) Checklist of Millipeds of North and Middle America. Virginia Museum of Natural History, Special Publication 8: 1–584.

[B17] Kuroda M Eguchi K Oguri E Nguyen AD (2022a) Two new cave *Hyleoglomeris* species (Glomerida, Glomeridae) from northern Vietnam. ZooKeys 1108: 161–174. 10.3897/zookeys.1108.85423 36760701 PMC9848618

[B18] Kuroda M Susukida M Sakamoto K Tsukamoto S Nguyen AD Oguri E Eguchi K (2022b) A new species of the genus *Hyleoglomeris* Verhoeff 1910 from Central Japan (Diplopoda: Glomerida: Glomeridae). Acta Arachnologica 71(2): 115–124. 10.2476/asjaa.71.115

[B19] Latreille PA (1802) Histoire naturelle, générale et particulière des Crustacés et des Insectes. Tome cinquieme. Dufart, Paris, 391 pp. 10.5962/bhl.title.15764

[B20] Leach WE (1815) A tabular view of the external characters of four classes of animals, which Linné arranged under Insecta; with the distribution of the genera composing three of these classes into orders, &c. and descriptions of several new genera and species. Transactions of the Linnean Society of London 11(2): 306–400. 10.1111/j.1096-3642.1813.tb00065.x

[B21] Liu W Golovatch S (2020) The first representatives of the millipede family Glomeridellidae (Diplopoda, Glomerida) recorded from China and Indochina. ZooKeys 954: 1–15. 10.3897/zookeys.954.54694 32821201 PMC7406547

[B22] Loomis HF (1943) New cave and epigean millipeds of the United States, with notes on some established species. Bulletin of the Museum of Comparative Zoology 92: 371–410.

[B23] Marek PE Bond JE (2006) Phylogenetic systematics of the colorful, cyanide-producing millipedes of Appalachia (Polydesmida, Xystodesmidae, Apheloriini) using a total evidence Bayesian approach. Molecular Phylogenetics and Evolution 41(3): 704–729. 10.1016/j.ympev.2006.05.043 16876439

[B24] Means JC Hennen DA Marek PE (2021) A revision of the minor species group in the millipede genus *Nannaria* Chamberlin, 1918 (Diplopoda, Polydesmida, Xystodesmidae). ZooKeys 1030: 1–180. 10.3897/zookeys.1030.62544 33958904 PMC8060247

[B25] Nguyen AD Nguyen SG Eguchi K (2021) A new *Rhopalomeris* species (Diplopoda: Glomerida: Glomeridae), and notes on the phylogenetic relationships between glomeridans in Vietnam. Zootaxa 4927(2): 257–264. 10.11646/zootaxa.4927.2.5 33756710

[B26] Nielsen M Margaryan A Nielsen TL Enghoff H Allentoft ME (2022) Complete mitochondrial genomes from museum specimens clarify millipede evolution in the Eastern Arc Mountains. Zoological Journal of the Linnean Society 196(2): 924–939. 10.1093/zoolinnean/zlac058

[B27] Oeyen JP Wesener T (2015) Steps towards a phylogeny of the pill millipedes: Non-monophyly of the family Protoglomeridae, with an integrative redescription of *Eupeyerimhoffiaarchimedis* (Diplopoda, Glomerida). ZooKeys 510: 49–64. 10.3897/zookeys.510.8675 PMC4523764 26257534

[B28] Racheboeuf PR Hannibal JT Vannier J (2004) A new species of the diplopod *Amynilyspes* (Oniscomorpha) from the Stephanian Lagerstätte of Montceau-les-Mines, France. Journal of Paleontology 78(1): 221–229. 10.1666/0022-3360(2004)078<0221:ANSOTD>2.0.CO;2

[B29] Rambaut A Drummond AJ Xie D Baele G Suchard MA (2018) Posterior summarisation in Bayesian phylogenetics using Tracer 1.7. Systematic Biology 67(5): 901–904. 10.1093/sysbio/syy032 29718447 PMC6101584

[B30] Recuero E Rodríguez-Flores PC (2020) A new Mediterranean species of *Dolistenus* (Diplopoda, Platydesmida, Andrognathidae), with an updated key for the genus and the first contribution for a barcode database of European Platydesmida. Zootaxa 4718(1): 123–133. 10.11646/zootaxa.4718.1.10 32230046

[B31] Reip HS Wesener T (2018) Intraspecific variation and phylogeography of the millipede model organism, the Black Pill Millipede *Glomerismarginata* (Villers, 1789) (Diplopoda, Glomerida, Glomeridae). ZooKeys 741: 93–131. 10.3897/zookeys.741.21917 30872937 PMC5904428

[B32] Shelley RM (2000) Annotated checklist of the millipeds of North Carolina (Arthropoda: Diplopoda), with remarks on the genus *Sigmoria* Chamberlin (Polydesmida: Xystodesmidae). Journal of the Elisha Mitchell Scientific Society 116: 177–205.

[B33] Shelley RM Golovatch SI (2011) Atlas of myriapod biogeography. I. Indigenous ordinal and supra-ordinal distributions in the Diplopoda: Perspectives on taxon origins and ages, and a hypothesis on the origin and early evolution of the class. Insecta Mundi 158: 1–134.

[B34] Silvestri F (1917) Contributions to a knowledge of the oriental DiplopodaOniscomorpha. I, The family Glomeridae. Records of the Indian Museum 13: 103–151. 10.26515/rzsi/v13/i3/1917/163604

[B35] Spelda J Reip HS Oliveira-Biener U Melzer RR (2011) Barcoding Fauna Bavarica: Myriapoda–a contribution to DNA sequence-based identifications of centipedes and millipedes (Chilopoda, Diplopoda). ZooKeys 156: 123–139. 10.3897/zookeys.156.2176 PMC3253575 22303099

[B36] Tamura K Stecher G Peterson D Filipski A Kumar S (2013) MEGA6: Molecular Evolutionary Genetics Analysis Version 6.0. Molecular Biology and Evolution 30(12): 2725–2729. 10.1093/molbev/mst197 24132122 PMC3840312

[B37] Trifinopoulos J Nguyen LT von Haeseler A Minh BQ (2016) W-IQ-TREE: A fast online phylogenetic tool for maximum likelihood analysis. Nucleic Acids Research 44(W1): W232–W235. 10.1093/nar/gkw256 PMC4987875 27084950

[B38] Vasquez-Valverde LF Marek PE (2022) Phylogenetic review of the millipede genus *Cherokia* Chamberlin, 1949 (Polydesmida, Xystodesmidae). ZooKeys 1106: 141–163. 10.3897/zookeys.1106.81386 36760818 PMC9848751

[B39] Verhoeff KW (1910) Über Diplopoden. 41. Aufsatz: Indomalayische Glomeriden. Sitzungsberichte der Gesellschaft Naturforschender Freunde zu Berlin 1910: 240–249.

[B40] Wesener T (2010) Revision of the American Pill Millipedes I: *Onomeris* and *Trichomeris* (Diplopoda, Glomerida, Glomeridae). Zootaxa 2725(1): 28–40. 10.11646/zootaxa.2725.1.2

[B41] Wesener T (2012) *Nearctomeris*, a new genus of Pill Millipedes from North America, with a comparison of genetic distances of American Pill Millipede Genera (Glomerida, Glomeridae). Zootaxa 3258(1): 58–68. 10.11646/zootaxa.3258.1.5

[B42] Wesener T (2015a) Integrative redescription of a forgotten Italian pill millipede endemic to the Apuan Alps-*Glomerisapuana* Verhoeff, 1911 (Diplopoda, Glomerida, Glomeridae). Zootaxa 4039: 391–400. 10.11646/zootaxa.4039.2.11 26624486

[B43] Wesener T (2015b) No millipede endemics north of the Alps? DNA-Barcoding reveals Glomeris malmivaga Verhoeff, 1912 as a synonym of *G.ornata* Koch, 1847 (Diplopoda, Glomerida, Glomeridae). Zootaxa 3999(4): 571–580. 10.11646/zootaxa.3999.4.7 26623596

[B44] Wesener T (2019) The oldest pill millipede fossil: a species of the Asiatic pill millipede genus *Hyleoglomeris* in Baltic amber (Diplopoda: Glomerida: Glomeridae). Zoologischer Anzeiger 283: 40–45. 10.1016/j.jcz.2019.08.009

[B45] Wesener T (2022) Integrative redescription of the enigmatic monotypic alpine pill millipede genus *Simplomeris* Verhoeff, 1936 (Glomerida, Glomeridae, Haploglomerinae). Zootaxa 5200(6): 550–564. 10.11646/zootaxa.5200.6.3 37045017

[B46] Wesener T Conrad C (2016) Local hotspots of endemism or artifacts of incorrect taxonomy? the status of microendemic pill millipede species of the genus *Glomeris* in Northern Italy (Diplopoda, Glomerida). PLoS One 11(9): e0162284. 10.1371/journal.pone.0162284 PMC5025202 27632210

[B47] Wilbrandt J Lee P Read H Wesener T (2015) A first integrative study of the identity and origins of the British Dwarf Pill Millipede populations, Trachysphaeracf.lobata (Diplopoda, Glomerida, Glomeridae). Biodiversity Data Journal 3: e5176. 10.3897/BDJ.3.e5176 PMC4493372 26175612

